# Disruption in the cecal microbiota of chickens challenged with *Clostridium perfringens* and other factors was alleviated by *Bacillus licheniformis* supplementation

**DOI:** 10.1371/journal.pone.0182426

**Published:** 2017-08-03

**Authors:** Yicen Lin, Shuai Xu, Dong Zeng, Xueqin Ni, Mengjia Zhou, Yan Zeng, Hesong Wang, Yi Zhou, Hui Zhu, Kangcheng Pan, Guangyao Li

**Affiliations:** 1 Animal Microecology Institute, College of Veterinary Medicine, Sichuan Agricultural University, Sichuan Province, Chengdu, China; 2 Key Laboratory of Animal Disease and Human Health of Sichuan Province, Sichuan Agricultural University, Sichuan Province, Chengdu, China; 3 Boshan Developing of Agricultural Technology Co., Ltd., Ya’an, China; University of New England, AUSTRALIA

## Abstract

*Clostridium perfringens* can induce necrotic enteritis of chickens, which causes large economic losses every year. *Bacillus licheniformis*, a probiotic, can inhibit the growth of pathogenic bacteria such as *Clostridium perfringens*, thereby improving the health status of chickens. However, from a microbial ecology perspective, the mechanisms by which alterations to the gut microbiota improve health remain unknown. In this study, we used Illumina MiSeq sequencing to investigate the cecal microbiota of a negative control group (NC), a *C*. *perfringens* and *Eimeria* challenge group with fishmeal supplementation (PC), a group supplemented with fishmeal and infected with coccidia (FC), and group PC with *B*. *licheniformis* supplementation (BL). We found that the health status of *C*. *perfringens*-challenged chickens was compromised, and that *B*. *licheniformis* improved the growth of the chickens challenged with pathogens. Microbial diversity analysis and taxonomic profiling of groups NC, PC, and FC revealed a disturbed cecal microflora of the birds with *C*. *perfringens*. We also characterized the microbiota of the chickens in the BL group using several methods. Principal coordinate analysis demonstrated that, compared with group PC, the bacterial community structure of group BL was more similar to that of group NC. Linear discriminant analysis with effect size revealed less differentially represented bacterial taxa between groups BL and NC than between groups PC and NC. In addition, groups BL and NC appeared to have similar overrepresented microbial taxa (such as *Bacteroides*, *Helicobacter*, *Megamonas*, and *Akkermansia*) compared with group PC. Finally, a phylogenetic investigation of communities by reconstruction of unobserved states analysis indicated that large differences existed between group PC and groups NC and BL. In conclusion, pre-treatment with *B*. *licheniformis* reduced the disturbance of the cecal microbiome induced by challenge with *C*. *perfringens* and other factors in broiler chickens.

## Introduction

*Clostridium perfringens* is a pathogen that leads to necrotic enteritis (NE) in broiler chickens [1], which has been estimated to cause financial losses of over $6 billion USD per year [2] and poses a huge risk to public health as a foodborne illness. However, *C*. *perfringens* infection alone is insufficient to cause clinical disease [3, 4]. A fishmeal diet and *Eimeria* infection are the most common predisposing factors, which work together with *C*. *perfringens* to cause NE in chickens [5–7]. Antibiotic growth promoters were used extensively to the control of *C*. *perfringens* infection. However, concerns about antimicrobial resistance and the potential for the overuse of antibiotics have caused many countries to restrict or ban the use of antibiotics for growth promotion [8]. Additionally, antibiotics can disturb the indigenous microflora. This microflora dysbiosis may expose animals and humans to other pathogens [9, 10].

Some alternatives to antibiotics, such as probiotics, prebiotics, essential oils, and organic acids, have been proposed [11]. A probiotic, defined as a bacterial species that benefits the host by improving the gut microbial balance [12], can inhibit harmful bacteria competitively [13]. Administration of spore-forming probiotic *Bacillus* spp. is one of the most promising approaches to preventing *C*. *perfringens* infection and NE outbreaks. Some studies have examined the effects of *Bacillus* spp. on the control of NE in broiler chickens. These studies have shown that dietary supplementation with *Bacillus* spp. is effective in mitigating the damage caused by *C*. *perfringens*. Most of these studies concluded that *Bacillus* spp. could increase growth in NE-infected chickens by promoting body weight gain (BWG) and feeding efficiency [14–17]. Importantly, treatment with *Bacillus* spp. elevated serum nitric oxide levels and decreased *coccidia*-specific antibodies relative to those in antibiotic-treated chickens [15]. Specifically, *Bacillus licheniformis* has been shown to prevent and treat NE [18–20]. Our previous study suggested that dietary *B*. *licheniformis* supplementation could enhance growth performance and antioxidant activity, as well as regulate fatty acid synthesis and oxidation-related genes in the livers of *C*. *perfringens*-challenged chickens [21].

The cecum, a multifunctional organ crucial to chicken physiology, allows chickens to digest starch, cellulose, and other polysaccharides. In addition, a normal microbiome can react to the invasion of pathogens and the presence of probiotics. Modern sequencing approaches have been used to explore the shift in the cecal microbiota caused by *C*. *perfringens* infection or other predisposing factors [22–24]. Nevertheless, there are few reports on the effects of probiotics on the microflora of chickens during the infection process.

Therefore, in this study, we examined cecal microbiota shifts in chickens challenged with *C*. *perfringens* and other factors following clinical treatment with *B*. *licheniformis*. Consistent with previous findings that probiotics could improve the health status and growth performance of chickens, we found that *B*. *licheniformis* treatment prevented disturbances in the cecal microflora in *C*. *perfringens* and other factors challenge chickens.

## Materials and methods

### Bacterial strains and *coccidia* vaccine preparation

In the present study, *B*. *licheniformis* H2 (CCTCC No.: M2011133) was obtained from the Animal Microecological Research Center in Sichuan Agricultural University, Chengdu, China. A type-A strain of *C*. *perfringens* (CVCC2030, from the China Institute of Veterinary Drug Control), originally isolated from a chicken diagnosed with clinical NE, was obtained from the China Veterinary Culture Collection Center. The defective leukemia virus coccidia attenuated vaccine was developed by Shanghai Veterinary Research Institute.

### Animal experiments

All animal experiments were conducted in compliance with the guidelines of the Sichuan Agricultural University Animal Care and Use Committee. The animal use protocol was approved and supervised by the Sichuan Agricultural University Animal Care and Use Committee. All animal manipulations were designed to reduce suffering.

Day-old broilers (n = 240) with similar body weights (45.35 ± 0.45 g) were divided randomly into four groups, with five replicates per treatment and 12 chicks per pen. Water and food were available *ad libitum* and light was constant throughout the experiment. The animal trial was conducted at the poultry house located in Sichuan Agricultural University.

Four groups were designated: a group fed a corn-soybean meal diet (negative control group, NC), an experimental group with *C*. *perfringens*, coccidia, and fishmeal (positive control group, PC), a group fed with 30% fishmeal as a supplement from day 14 and inoculated with coccidia on day 15 (fishmeal and coccidia group, FC), and a *C*. *perfringens*, coccidia challenge and fishmeal supplementation group treated with a *B*. *licheniformis* supplement (1.0 × 10^6^ colony-forming units (CFU)/g, BL group).

The birds were fed a typical corn-soybean diet from days 1 to 13. The compositions of unmedicated corn-soybean meal and high fishmeal diets are shown in S1 Table. From day 14, all groups except the NC group were fed basal diets supplemented with 30% fishmeal (weight/weight). On day 15, chickens from all groups except the NC group were treated with a 10-fold coccidia vaccine via oral gavage. In the NC group, sterile phosphate-buffered saline was administered instead. On days 18, 19, and 20, birds in the PC and BL groups were given 1 mL of *C*. *perfringens* (approximately 2.2 × 10^8^ CFU/mL) through a plastic tube, whereas the others were treated with sterile physiological saline. The feed had first been mixed with *B*. *licheniformis* bacterial suspensions, then given to the chickens in the BL group at a dose of 1.0 × 10^6^ CFU/g throughout the animal trial. Cecal samples were collected on day 22.

Animals were monitored twice daily until the pathogens were administered, then three to four times daily thereafter. Several chickens succumbed to sudden death overnight in the *C*. *perfringens*-infected groups, although they had not previously been severely ill. To reduce the stress on the chickens, they were treated more carefully and isolated from staff and animals that were unrelated to the study. Veterinary care was provided, which increased the monitoring of the animals. We also increased the frequency of the cleaning of the animal housing in response to environmental and behavioral factors. Given that the chickens in group PC had clinical symptoms and died suddenly overnight, we were unable to perform euthanasia on the birds. Four chickens from group PC died from *C*. *perfringens* treatment.

### Sample collection

On day 22, 60 birds were selected randomly and sacrificed by cervical dislocation (15 birds from each treatment group, including three from each replicate); we extracted their cecal content by dissection. The cecal content was immediately placed at −80°C and stored until the DNA was extracted.

### DNA extraction from cecal contents

DNA was isolated using an E.Z.N.A.^®^ Stool DNA Kit (Omega, USA), according to the manufacturer’s manual. The isolated DNA was divided into aliquots and stored at −80°C until sequencing.

### Illumina MiSeq sequencing procedure

Illumina MiSeq sequencing was performed at the Chengdu Institute of Biology, Chinese Academy of Sciences. Briefly, DNA samples were amplified using the primer set 515F/806R (515F: 5′-GTGCCAGCMGCCGCGGTAA-3′, 806R: 5′-AACGCACGCTAGCCGGACTACVSGGGTATCTAAT-3′), which targets the V4 region of the bacterial 16S rDNA. The polymerase chain reaction (PCR; total volume, 100 μL) comprised 0.75 units of Ex Taq^®^ DNA polymerase (Takara, Dalian, China), 1× Ex Taq^®^ loading buffer (Takara), 0.2 mM dNTP mix (Takara), 0.2 μM of each primer, and 100 ng of template DNA. Thirty-five PCR cycles were performed (95°C for 3 min, 94°C for 100 s, 50°C for 1 min, 72°C for 1 min, and 72°C for 10 min). Finally, as described by Caporaso et al. [25], pyrosequencing was performed on an Illumina MiSeq 2 × 250 platform.

### Sequencing data analysis

After sequencing, whole reads were assembled using the mothur software, then the chimeric sequences were removed based on the UCHIME algorithm in USEARCH software [26]. Clean data were analyzed using QIIME software, and operational taxonomic units (OTUs) were determined based on a 97% similarity threshold. Alpha diversity measures, including the Shannon, observed OTU, and Chao1 indices, were investigated in QIIME, and the significance of these estimates was determined using a Mann–Whitney U test. In addition, jackknifed beta diversity (with 10 resamplings) was calculated from unweighted and weighted UniFrac distances, and a 3D principal coordinate analysis (PCoA) was generated in QIIME [27]. A two-sided Student’s *t*-test was used to determine the significance of the differences between groups.

Linear discriminant analysis with effect size (LEfSe) [28] and phylogenetic investigation of communities by reconstruction of unobserved states (PICRUSt) [29] were performed online (http://huttenhower.sph.harvard.edu/galaxy/), based on the taxonomic files obtained from the QIIME analysis. The R packages “pheatmap,” “Phyloseq,” and “biom” were used for data analysis and image plotting [30, 31]. The raw read sequences of our 60 samples have been deposited at the Sequence Read Archive of the National Center for Biotechnology Information, with the study accession number SRP094630.

## Results

### Pathological changes and growth performance in broiler chickens challenged with *C*. *perfringens*

The mortality rate of the chickens in group PC after *C*. *perfringens* treatment was 6.7% (4/60). After dissection, pathological examination of the deceased broilers in group PC revealed that *C*. *perfringens* had caused great damage to the chickens. The dissected broiler chickens presented with clinical symptoms (S1 File).

The mean values of the growth performance parameters from day 1 to 22 are shown in Table 1. The BWG of the chickens in group PC was significantly lower than that in group NC (*P* < 0.05). The feed conversion ratio (FCR) of group PC was also significantly lower than that of group NC (*P <* 0.05). However, the BWG of the chickens in group BL was higher than that of group PC, and not significantly different from that of group NC (*P* > 0.05).

**Table 1:** Effects of *Bacillus licheniformis* on the growth performance of broilers from day 1 to 22.

	NC	PC	FC	BL
BWG (g)	751.33 ± 20.43^a^	659.00 ± 22.19^b^	694.80 ± 22.13^ab^	711.67 ± 15.87^ab^
FI (g)	1125.61 ± 12.16	1119.38 ± 16.21	1102.69 ± 13.40	1129.21 ± 17.24
FCR (g/g)	1.50 ± 0.05^b^	1.70 ± 0.06^a^	1.59 ± 0.05^ab^	1.59 ± 0.02^ab^

Across rows, lowercase letters indicate statistically significant differences (*P <* 0.05). NC, negative control; PC, treated with *C*. *perfringens*, coccidia, and fishmeal; FC, treated with fishmeal and coccidia; BL, pre-treated with *B*. *licheniformis* then treated with *C*. *perfringens*, coccidia, and fishmeal; BWG, body weight gain; FI, feed intake; FCR, feed conversion ratio

### The cecal bacterial community was disturbed by *C*. *perfringens* together with predisposing factors

The difference between groups PC and FC was that the former were fed with *C*. *perfringens*. This part of the study was designed to determine the impact of *C*. *perfringens* on the cecal microbiome in combination with predisposing factors (fishmeal feeding and coccidia infection).

We calculated several alpha diversity indexes to estimate species diversity and richness in QIIME for groups PC, FC, and NC. These metrics included the Shannon, observed species, and Chao1 indices. The index values and significant differences between samples and groups are shown in S1 Fig. The Shannon index results indicated that *C*. *perfringens* infection with predisposing factors caused greater changes in microbial diversity than those observed in the negative control (Panel A in S1 Fig, *P* < 0.01). Predisposing factors alone also significantly increased the species diversity (Panel A in S1 Fig, *P* < 0.05). The observed species and Chao1 indices indicated the species richness of the bacterial community. As shown in panel B and panel C in S1 Fig, the species richness in group PC did not differ statistically from that of group NC. However, predisposing factors alone, without *B*. *licheniformis* treatment, increased the microbial richness significantly compared with that of group NC (*P* = 0.007 for the observed species index and *P* = 0.01 for the Chao1 estimate).

A beta diversity analysis was used to assess the microbial structure of each sample. A PCoA allows visualization of the relationships between the bacterial community structures of several samples based on unweighted and weighted UniFrac distances. The two-axis plots of three groups (NC, FC, and PC) are presented in S2 Fig. Groups FC and PC showed greater disorder, based on within-group distances, than group NC. Unsurprisingly, the samples from group PC were distinct from those of group NC. In addition, we determined within-group and between-group unweighted UniFrac distances. The unweighted UniFrac distances are shown in S2 Table. Interestingly, the between-group comparison for all three groups revealed that their beta diversities differed significantly.

After the microbial diversity analysis, we explored the taxonomic profiles of the three groups. We identified 162 bacterial taxa at the genus level in the three groups (S3 Fig). The number of unique bacterial taxa in each group was 37 (PC), seven (FC), and 10 (NC). Notably, among the groups, group PC had the highest number of unique bacterial taxa, which represented 22.8% of all taxa detected in the three groups.

### Characterization of the cecal microbiota in the chickens that received *B*. *licheniformis*

*B*. *licheniformis* has been shown to reduce the harmful effects of *C*. *perfringens* and NE-predisposing factors in chickens. However, rather than a traditional perspective, in this study, we present ecological insights into the preventative treatment.

**Fig 1:**
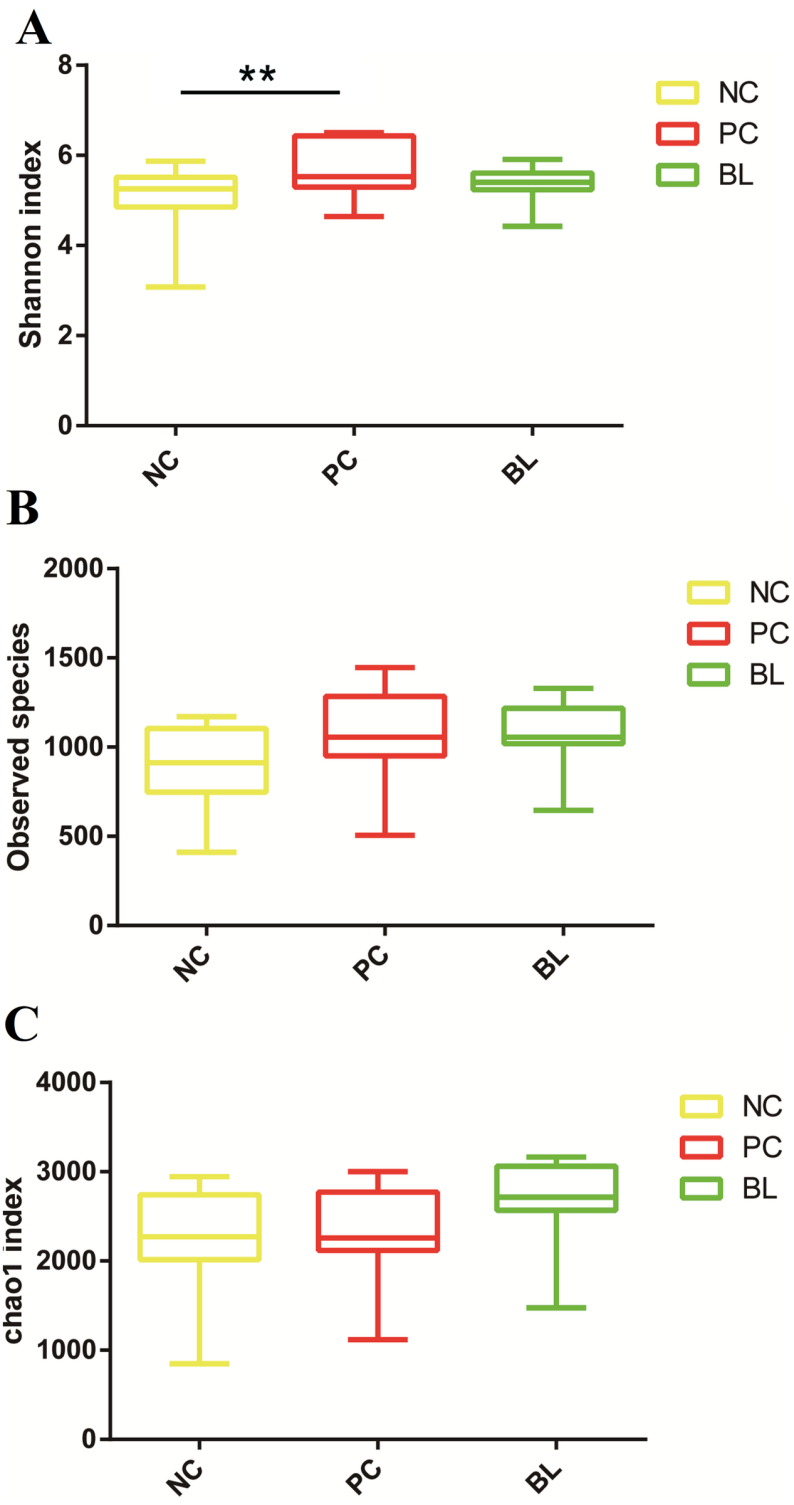
Effects of *C*. *perfringens* with other factors and *Bacillus licheniformis* treatment on microbial diversity and richness in chickens. Boxplots depicting the alpha diversity indexes Shannon (A), Observed Species (B) and chao1 (C). *, *P* < 0.05; **, *P* < 0.01 by Mann–Whitney U test. NC, negative control; PC, treated with *C*. *perfringens*, coccidia, and fishmeal; BL, pre-treated with *B*. *licheniformis* then treated with *C*. *perfringens*, coccidia, and fishmeal.

We first determined the alpha diversity in the *B*. *licheniformis* treatment group (group BL), and then investigated its differences compared with groups NC and PC in QIIME. Surprisingly, the results showed that when all three indexes were considered, there were no significant differences in the alpha diversity measures in group BL and groups NC or PC (Fig 1).

**Fig 2:**
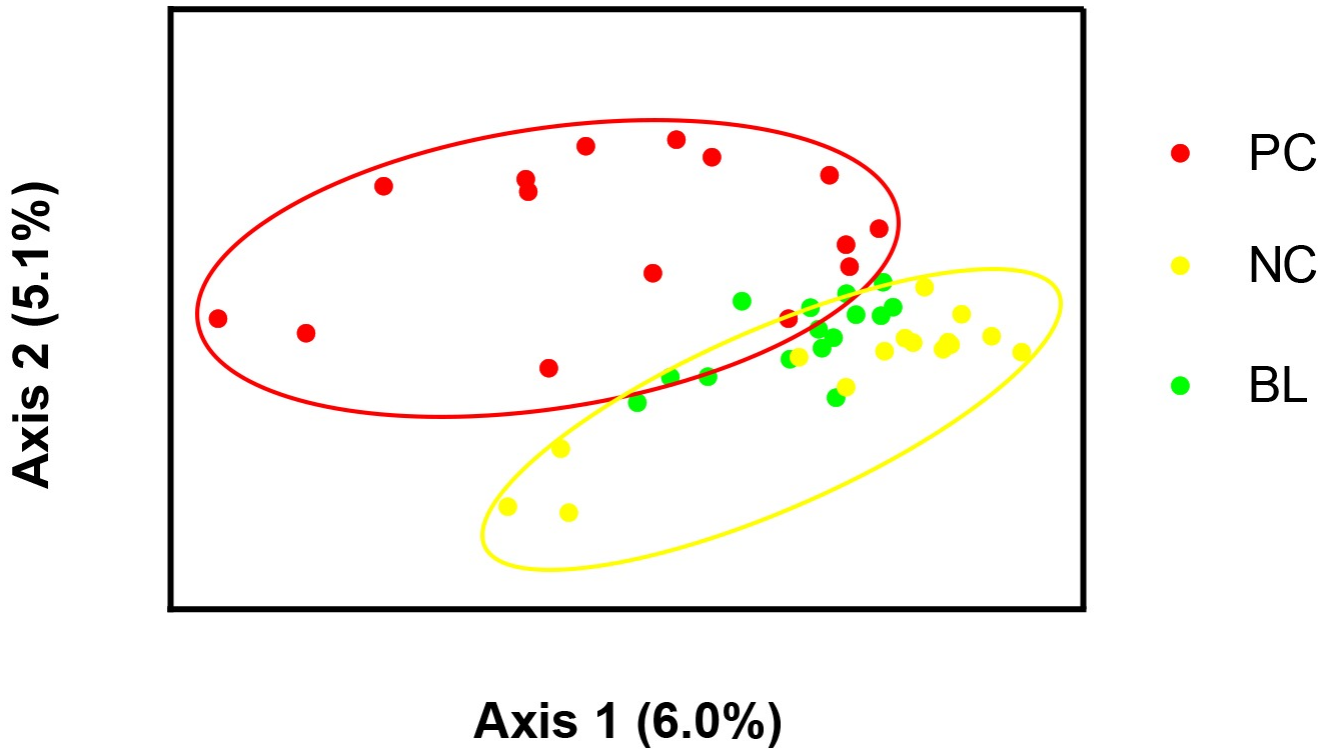
Principal coordinate analysis of the bacterial community structures in the cecal microbiota of chickens with *C*. *perfringens* in the presence and absence of *Bacillus licheniformis* treatment. NC, negative control; PC, treated with *C*. *perfringens*, coccidia, and fishmeal; BL, pre-treated with *B*. *licheniformis* then treated with *C*. *perfringens*, coccidia, and fishmeal.

A PCoA plot was constructed to assess the relationships between the community structures of three of the groups (NC, PC, and BL). As shown in Fig 2, samples from group BL were clustered and were more similar to those of group NC, whereas the samples from the group PC were dispersed. These results indicated that the bacterial community disturbance caused by *C*. *perfringens* infection was alleviated by *B*. *licheniformis* treatment.

**Fig 3:**
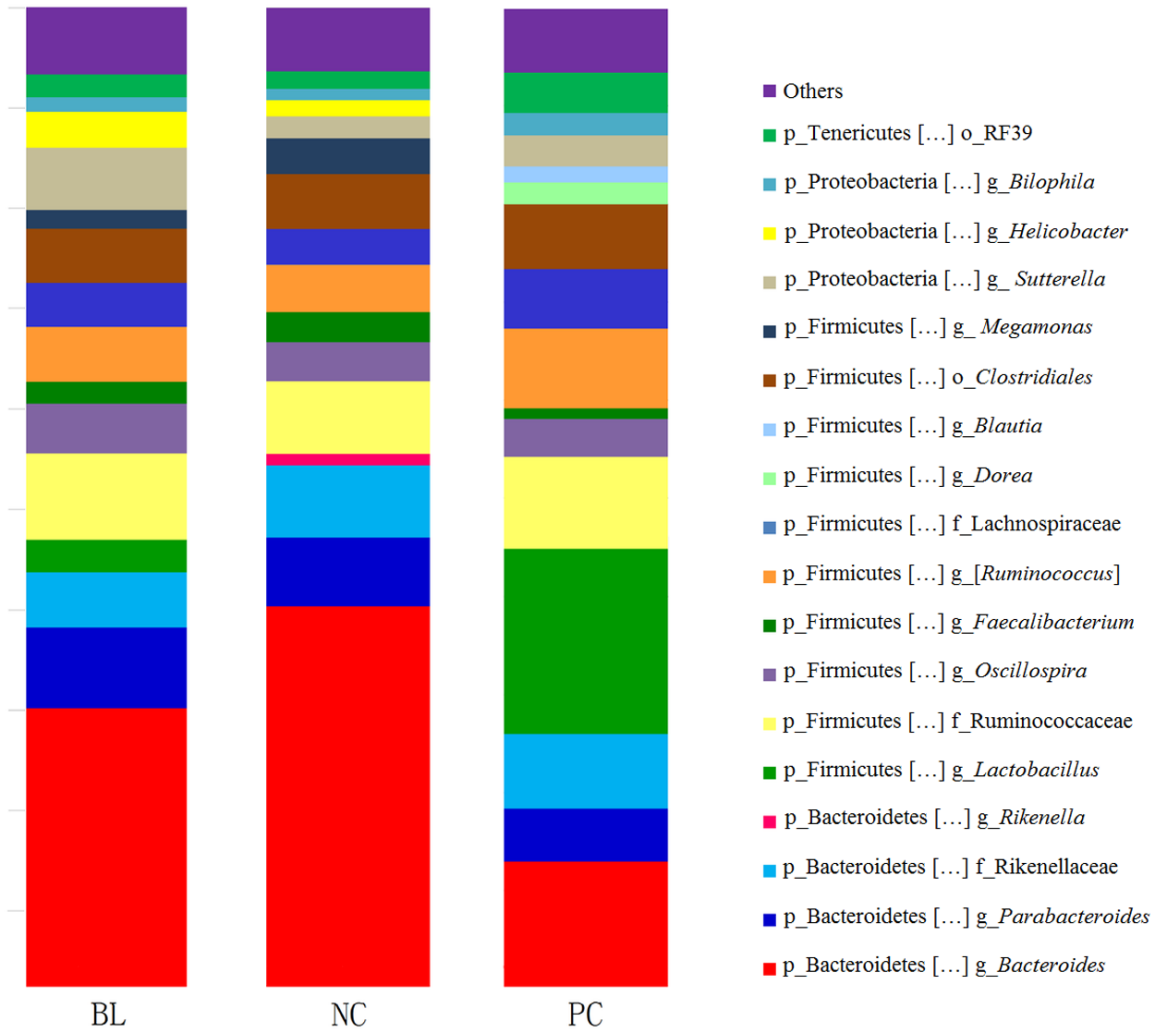
Cecal microbial composition of chickens with *C*. *perfringens*, *Eimeria* challenge and fishmeal supplementation. The 15 most abundant taxa in each group are shown to the phylum or genus level. Sequences that could not be classified at the genus level were labeled with the next highest level. NC, negative control; PC, treated with *C*. *perfringens*, coccidia, and fishmeal; BL, pre-treated with *B*. *licheniformis* then treated with *C*. *perfringens*, coccidia, and fishmeal.

To determine the most abundant taxa in the three groups and the taxonomic differences between them, we analyzed the 15 most abundant taxa at the genus level in the three groups (Fig 3). The relative abundances of these genera in a total of 45 samples, and the standard deviation of each group, are included in S3 Table. Analysis of the 15 most abundant taxa showed that all groups comprised approximately the same community profiles (the three groups shared 12 common taxa). However, the relative abundances of these taxa varied between the groups. Notably, the relative abundance of *Bacteroides* in group NC was the highest among the three groups. The abundance of *Bacteroides* spp. was the lowest in group PC. The lowest relative abundance of *Bacteroides*, which are well-known beneficial bacteria, might indicate a disturbance of the microflora in group PC. In addition, the bacterial community profiles of group PC were different from those of the other two groups. One of the most surprising findings was that the mean value of the relative abundance of *Lactobacillus* in group PC was significantly higher than that in the other groups, with *Lactobacillus* even ranking as the most abundant taxon in some samples.

**Fig 4:**
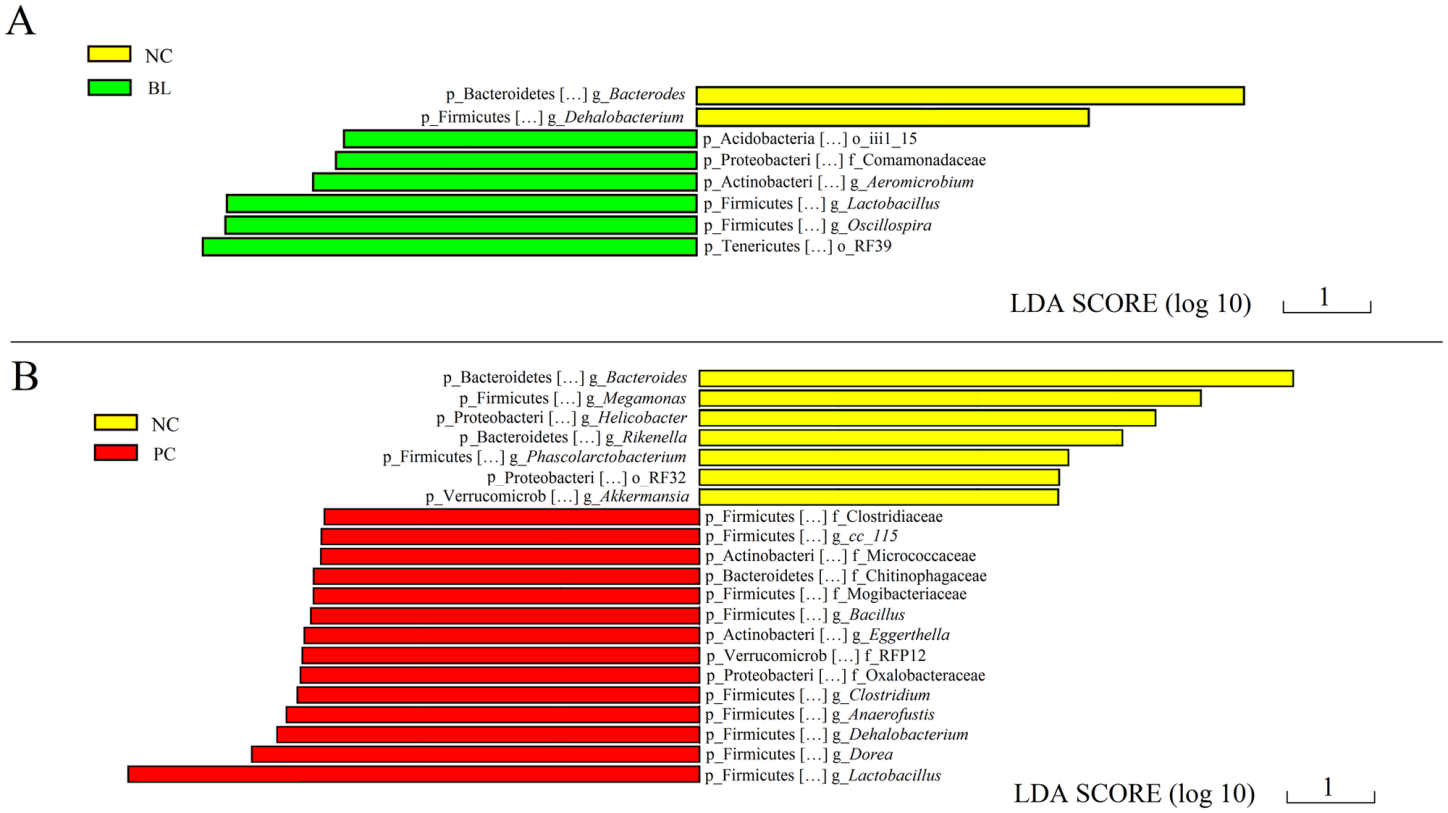
Taxa that were significantly differentially represented in the control group compared with the groups that had *C*. *perfringens*, *Eimeria* challenge and fishmeal supplementation, with and without *Bacillus licheniformis* treatment. **(**A) Differentially overrepresented taxa in group NC versus group BL. (B) Differentially overrepresented taxa in group NC versus group PC. Phylum and genus level names are shown. Sequences that could not be classified at the genus level were named according to the next highest level. NC, negative control; PC, treated with *C*. *perfringens*, coccidia, and fishmeal; BL, pre-treated with *B*. *licheniformis* then treated with *C*. *perfringens*, coccidia, and fishmeal.

**Fig 5:**
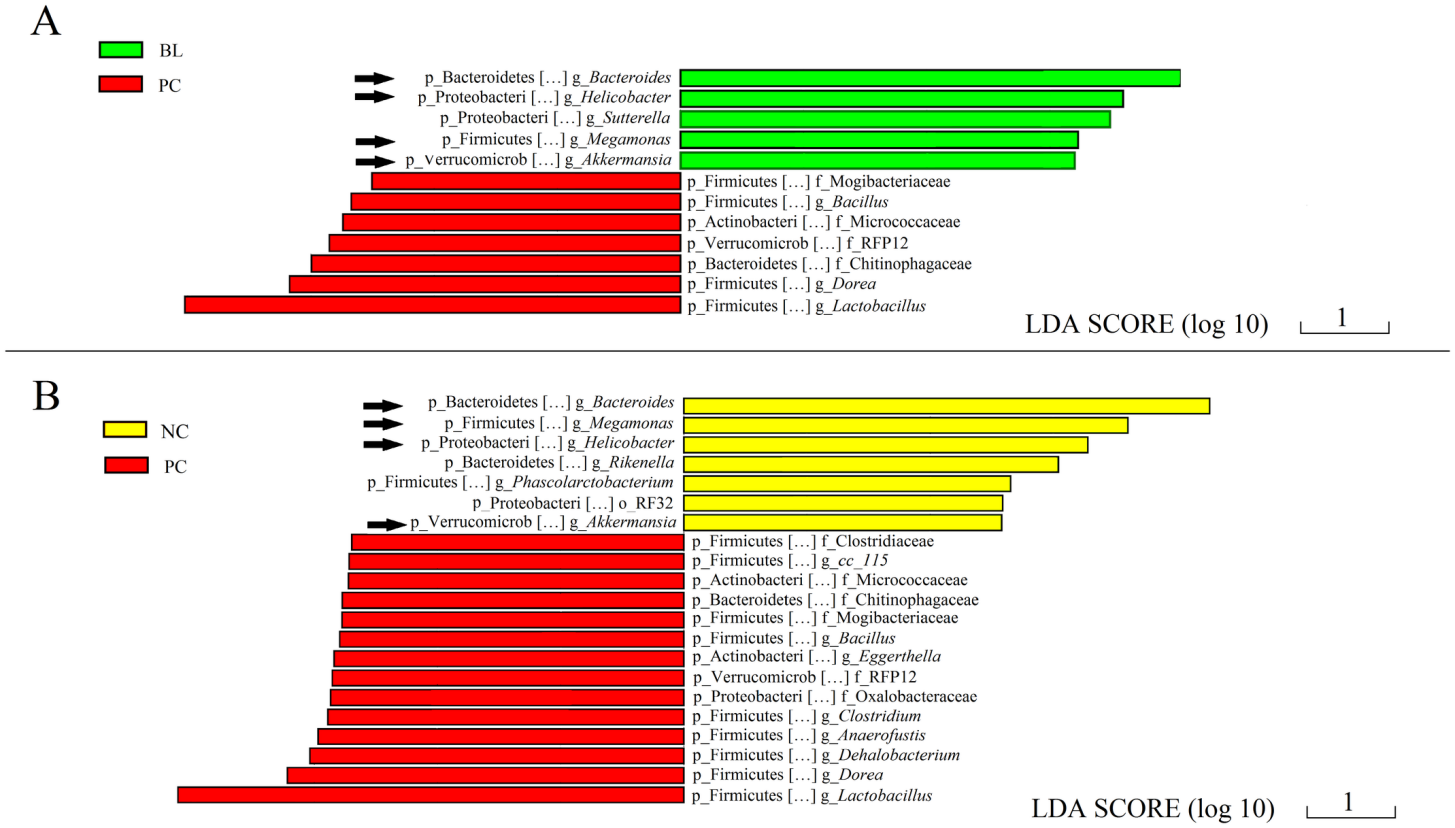
Taxa that were significantly differentially represented in the ceca of chickens with *C*. *perfringens*, *Eimeria* challenge and fishmeal supplementation compared with the negative control and *Bacillus licheniformis* treatment groups. **(**A) Differentially overrepresented taxa in group PC versus group BL. (B) Differentially overrepresented taxa in group PC versus group NC. Phylum and genus level names are shown. Sequences that could not be classified at the genus level were named according to the next highest level. NC, negative control; PC, treated with *C*. *perfringens*, coccidia, and fishmeal; BL, pre-treated with *B*. *licheniformis* then treated with *C*. *perfringens*, coccidia, and fishmeal.

To fully understand the effects of *B*. *licheniformis* treatment, it is crucial to evaluate the specific taxonomic differences among the groups. We used LEfSe to identify the taxa that were significantly different between the groups. LEfSe is an algorithm designed to identify the microbial taxa that contribute to differences between two or more environmental samples. In our study, taxa with an average relative abundance over 0.0001 were selected for LEfSe. This threshold ensured that meaningful taxa were retained and the rarest taxa were removed. Pair-wise comparisons among the three groups were made using LEfSe. The characteristics of these comparisons are presented in Figs 4 and 5.

As shown in Fig 4, we performed two comparisons using LEfSe (group NC versus group BL in Fig 4A, and group NC versus group PC in Fig 4B). There were more differentially represented bacterial taxa in the comparison of group NC with group PC; fourteen bacterial taxa were overrepresented in the *C*. *perfringens* infection group (PC), whereas another seven taxa were significantly more abundant in group NC. We speculated that there would be fewer specific taxonomic differences between groups NC and BL.

Fig 5A shows the differentially represented taxa between groups PC and BL, whereas Fig 5B shows those between groups PC and NC. Compared with group PC, several bacterial taxa (*Bacteroides*, *Helicobacter*, *Megamonas*, and *Akkermansia*) were overrepresented significantly in groups BL and NC. Seven bacterial taxa were overrepresented in group PC compared with group BL (Fig 5A), and these seven bacterial taxa were also overrepresented in group PC compared with group NC (Fig 5B).

These findings may indicate that groups NC and BL share specific taxonomic similarities.

**Fig 6:**
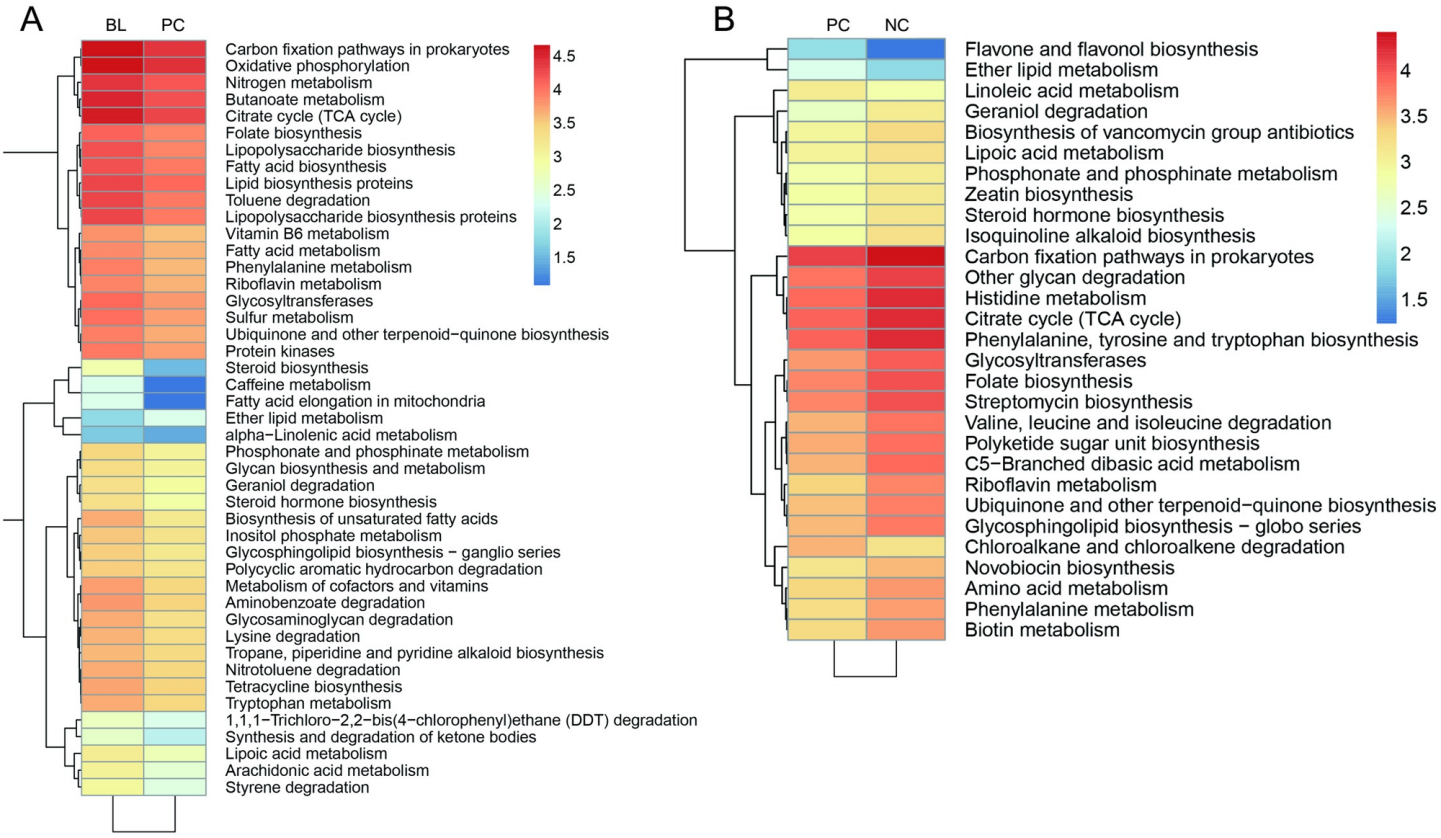
Predicted functional profiles of the cecal microbiota of control chickens and those with *C*. *perfringens*, *Eimeria* challenge and fishmeal supplementation, in the presence and absence of *Bacillus licheniformis*. Differences between groups were assessed using a bootstrap Mann–Whitney U-test with cutoffs of *P* < 0.01, false discovery rate < 0.1, and mean counts >10. NC, negative control; PC, treated with *C*. *perfringens*, coccidia, and fishmeal; BL, pre-treated with *B*. *licheniformis* then treated with *C*. *perfringens*, coccidia, and fishmeal.

PICRUSt allowed us to predict functional profiles from the taxonomic profiles. Using the Kyoto Encyclopedia of Genes and Genomes (KEGG) database of microbial genomic information, we identified significant differences in the OTUs (*P* < 0.01) in the groups, and performed a PICRUSt analysis online. This analysis allowed a comparison of the differences in the functional profiles of groups PC and BL, and revealed gene pathways that were significantly different between groups NC, PC, and BL. We found that, although only 14 gene pathways differed between groups BL and NC, 76 gene pathways differed between groups PC and BL, and 57 differed between groups NC and PC (S2 File). This may be explained by the fact that the functional profiles representing the microbial communities in groups BL and PC were relatively distinct. Of the KEGG pathway types, the most notable were the metabolism gene pathways. After filtering, 45 out of the 76 pathways differing between groups PC and BL, and 29 out the 57 between groups NC and PC, were confirmed as related to metabolism (Fig 6). Specifically, 44 metabolic pathways were more abundant in group BL than in group PC, but only one metabolic pathway was more abundant in group PC. A similar trend was found between groups NC and PC. Four metabolic pathways were enriched in group PC compared with NC, whereas 25 metabolic pathways were more abundant in group NC. Furthermore, the metabolic pathways included those for glycans, lipids, and amino acids, as well as xenobiotic metabolism.

## Discussion

### The harmful effects of *C*. *perfringens* and the growth-promoting effects of *B*. *licheniformis*

Before the analysis of the sequencing data, it was necessary to evaluate the effects of *C*. *perfringens* and *B*. *licheniformis* on broilers. *C*. *perfringens* caused damage to the chickens, by reducing their growth performance and causing sudden death. The damaging effects of *C*. *perfringens* have been well-documented [32]. By contrast, *B*. *licheniformis* mediated pro-growth effects. These results provided the background for the further bacterial community analysis.

### Disturbance in the cecal microflora of chickens following the *C*. *perfringens*, *Eimeria* challenge and fishmeal supplementation

Occasionally, even in the absence of *C*. *perfringens* infection, improper management of poultry, such as providing high protein feed and leaving parasite infections unchecked, can result in economic losses. It is well established that *C*. *perfringens* challenge is associated with altered microflora community structures [22], and differential responses to the three inducing factors (fishmeal, *Eimeria*, and *C*. *perfringens*) have been evaluated in an NE model [23]. However, the specific differences, including bacterial community structure and composition, in the absence or presence of *C*. *perfringens* with predisposing factors have not been thoroughly examined. To determine these differences, we investigated the community structures and taxa present in groups PC and FC.

With regard to the alpha diversity of the cecal microbiota, our findings suggested that predisposing factors, even without *C*. *perfringens*, could increase microbial diversity significantly (Shannon index) compared with that in group NC. However, the additional interfering factors (*C*. *perfringens* in combination with predisposing factors) in the PC group induced greater changes to the alpha diversity. The observed species and Chao1 indices revealed that predisposing factors alone could affect species richness significantly. Species richness relies only on a count of species and does not take into account the relative abundance of these species. Although the mechanisms underlying the interactions between *C*. *perfringens* and predisposing factors are not well understood, there is a popular theory that these factors can disturb the cecal environment, providing nutrients or preferential ecological niches for the proliferation of *C*. *perfringens* [3]. When *C*. *perfringens* was administered in the context of predisposing factors, the nutrients and niches provided could be utilized by *C*. *perfringens*. However, predisposing factors alone provided an opportunity for the proliferation of previously absent microorganisms. Therefore, the species richness of group FC was significantly higher than that of groups NC and PC. Contrastingly, *C*. *perfringens* infection might inhibit the proliferation of minor components of the microbiota [33], which could explain why there were no significant differences between the observed species and Chao1 indices for groups PC and NC.

The PCoA indicated that the samples from group PC were distinct from those of group NC, which is consistent with the findings of a previous study [22]. Whereas groups PC and NC showed distinct microbiota, samples from the FC group were not completely separate from those of either of the other two groups. This might be explained by the fact that the bacterial community structure of group FC, though not completely distinct from that of groups PC and NC, was also disturbed by the predisposing factors. The Venn diagram (S3 Fig) also showed distinct microbiota in the chickens in groups PC and NC.

### *B*. *licheniformis* treatment prevented the disturbance caused by *C*. *perfringens*, *Eimeria* challenge and fishmeal supplementation in the cecal microbiota

Although probiotics such as *B*. *licheniformis* have been applied previously to prevent the harmful effects of *C*. *perfringens* challenge in broilers, this is the first time that the intestinal microbiota of the prevention group has been analyzed from an ecological perspective.

There were no significant differences between the BL and the PC or control groups in the three alpha diversity indices for the microbial communities. *C*. *perfringens*, *Eimeria* challenge and fishmeal supplementation is associated with changes in microbial diversity in chicken ceca; the lack of an apparent difference between the diversity in groups BL and NC might imply that, after probiotic pre-treatment, *C*. *perfringens* and predisposing factors were unable to induce disorder in the microbial communities, which would be reflected in the alpha diversity measures. However, the UniFrac distance analysis suggested that the microbial structure of group BL was similar to that of group FC. This result is interesting, because it reflects the fact that, after administration of *B*. *licheniformis*, *C*. *perfringens* could not alter the bacterial community structure significantly and that only the predisposing factors affected the cecal microbiota. We hypothesized that this was because of inhibition of *C*. *perfringens* by *B*. *licheniformis*. Although the specific mechanism has not been determined, many studies have demonstrated the inhibitory capacity of *Bacillus* spp. [19, 20].

The top 15 taxa of groups NC, BL, and PC, however, indicated disorder in the microbiota. In studies of normal chickens, cecal communities are mainly colonized by the phyla Firmicutes, Bacteroides, and Proteobacteria [34–36], which agrees with the results of our study. At the genus level, however, there is controversy over the taxa that are predominant in chicken ceca. For instance, in a study by Callaway et al. [37], the most abundant genera were *Bacteroides* and *Prevotella*, whereas Stanley et al. [38] found that *Ruminococcus*, *Lactobacillus*, and *Bacteroides* were predominant. These discrepancies may reflect differences in the hosts, feeding regimens, and analytical techniques [36]; hence, it would be inappropriate to define microbiomes in chicken ceca as “healthy” or “unhealthy”. In our study, one of the most interesting findings is that *C*. *perfringens*, administered with fishmeal and coccidia, increased the relative abundance of *Lactobacillus* significantly, such that it became the most abundant taxon in some samples. Considering that the number of replicates was large (n = 15), this discovery about the cecal microbial chaos might be meaningful. *Lactobacillus*, a widely used probiotic, inhibits the growth and colonization of *C*. *perfringens* via the production of organic acids or bacteriocins [39–41]. Nevertheless, in this experiment, an increase in *Lactobacillus* resulted from infection with *C*. *perfringens*. Although this result was unexpected, some previous studies have shown similar results. Research on three breeds of commercial broilers with NE revealed that, compared with controls, the proportion of *Lactobacillus* in the bacterial community increased with NE in Cobb chickens but decreased in Ross and Hubbard chickens [42]. A study in 2012, which examined experimental NE in Arbor Acre broilers, found higher levels of lactobacilli in the guts of chickens with NE compared with that in the controls [43]. In our research, the mean proportion of *Lactobacillus* in the group with NE was significantly higher than that in the other three groups. It was not possible for us to determine a consistent pattern in the changes of the *Lactobacillus* population. Several studies have concluded that the *Lactobacillus* fraction is not dependent on NE status or the *C*. *perfringens* dose [44, 45]. However, the present study showed that *C*. *perfringens* together with predisposing factors could cause larger perturbations in the microbiota in chicken ceca in the absence of supplementation with *B*. *licheniformis*.

LEfSe indicated that there were smaller differences between the NC and BL groups than between the BL and PC groups with regard to the number of significantly overrepresented bacterial taxa. This finding supports the notion that the disturbances caused by *C*. *perfringens* could be alleviated by treatment with *B*. *licheniformis*. The chickens in groups BL and NC seemed to have similar overrepresented bacterial taxa, which could also support the preventative effects of *B*. *licheniformis*. Most importantly, these bacterial taxa might play a crucial role in the alleviation of the disturbance of the microbiota.

Notably, compared with in the other two groups, the relative abundances of *Lactobacillus* and *Dorea* were significantly higher in group PC, which may indicate that these taxa are also important biomarkers of *C*. *perfringens*, *Eimeria* challenge and fishmeal supplementation chickens. Other studies have obtained similar results. For example, in a study on Crohn’s disease, several genera, including *Dorea* and *Lactobacillus*, were more abundant in the colons of patients with Crohn’s disease than in patients without inflammatory bowel disease [46]. *Dorea* has great saccharolytic potential; in a study on children with irritable bowel syndrome, *Dorea* was overrepresented in the group treated with a low-dose fermentable oligosaccharides, disaccharides, monosaccharides, and polyols diet, and it is believed that this diet could result in decreased microbial fermentation [47]. Therefore, it was hypothesized that, with a decrease in microbial fermentation, taxa with strong saccharolytic metabolic capacity, such as *Dorea*, become more active. In our study, the relative abundance of *Dorea* was significantly higher than that of other genera, which suggested that *C*. *perfringens*, *Eimeria* challenge and fishmeal supplementation affected fermentation in the ceca. The cecal microbiota of groups BL and NC showed similar overrepresented taxa compared to those of group PC, based on the LEfSe. These taxa included *Bacteroides*, *Helicobacter*, *Megamonas*, and *Akkermansia*. The decrease in *Bacteroides* with *C*. *perfringens*, *Eimeria* challenge and fishmeal supplementation was a novel finding. Many studies have examined *Bacteroides*, and this taxon is considered an important contributor to the intestinal health of animals. It has also been defined as a probiotic that is beneficial for weight gain and growth performance [48–50]. The fermentation products of *Bacteroides* inhibit sporulation of *C*. *perfringens*, and depletion of the genus *Bacteroides* may predispose the animal gut to *C*. *perfringens* infection and gastroenteritis [51]. To the best of our knowledge, this is the first report of a probiotic treatment resulting in an increase in the abundance of *Bacteroides* in the ceca of chickens with *C*. *perfringens*, *Eimeria* challenge and fishmeal supplementation. This probiotic treatment could restore the microbial community to its normal status and improve the health of the chickens.

*Megamonas* and *Helicobacter* together were confirmed to be hydrogen sinks in the chicken cecum. The higher abundance of these two taxa can be explained partly by their ability to remove hydrogen, which could benefit the other members of the microbial community, as well as improve the host’s ability to recover energy from food [52]. The significant decrease in these two taxa in group PC might indicate an effect on the potential for energy recovery. More importantly, *Akkermansia*, described as a wound-mucosa-associated microbiota, could contribute to the enhanced repair of mucosal wounds [53]. The overrepresented taxa Akkermansia in group BL indicated the improved intestinal health compared with group PC.

Other taxa should also be explored. *Sutterella*, for example, is a potential probiotic present in pigeon “milk” that can improve the rate of growth and feed conversion ratio in chickens [54].

In the PICRUSt analysis, we found larger differences between PC and BL, than between the PC and NC groups (76 and 57 significantly different pathways, respectively). After screening, we found that metabolic pathways were the most common among the significantly differentially represented pathways, which could explain the large metabolic differences between group PC and the other two groups. We also investigated the KEGG pathway types and found that many metabolic pathways associated with lipids (12 out of 45) were shared between groups PC and BL, which agrees with a former study on the expression of lipid metabolism-related genes in broilers with *C*. *perfringens*, *Eimeria* challenge and fishmeal supplementation that were treated with *B*. *licheniformis* [21]. Although the expression of lipid metabolism-related genes was not associated with the microbiome in each group, we speculated that changes in the microbiota caused by *B*. *licheniformis* treatment played a positive role in the expression of lipid metabolism-related genes. Another KEGG pathway type, glycan metabolism, also warrants further study. This study showed that *C*. *perfringens*, *Eimeria* challenge and fishmeal supplementation leads to the destruction of glycan metabolism in group PC.

Does all of this indicate that *B*. *licheniformis* had a positive influence on the cecal microbiota of chickens with *C*. *perfringens*, *Eimeria* challenge and fishmeal supplementation? Given that large differences between individual chickens of the same breed or experimental trial have been reported, even in carefully controlled conditions [55], it is impossible to define “good” or “bad” microbiota in the chicken cecum. Nevertheless, based on this trial, the microbial communities of normal chickens appeared relatively healthy. The cecal microbiota of group BL chickens was more similar to that of chickens in group NC; therefore, we concluded that disruption of the cecal microbiota of chickens with *C*. *perfringens*, *Eimeria* challenge and fishmeal supplementation was alleviated by *B*. *licheniformis* supplementation.

## Supporting information

S1 Table Diet composition and nutrient levels. (DOCX)

S2 Table Significant within-group and between-group differences. (XLSX)

S3 Table Relative abundances of 15 bacterial genera in 45 samples from the negative control (NC), PC, and BL treatment groups. (XLSX)

S1 File Lesions induced by *Clostridium perfringens* on the mesentery and luminal surface of the small intestines of chickens. Large focal lesions were found on the luminal surfaces of the chickens’ small intestines (pictures A and B). We also found congestion of the mesentery (picture C).
(DOCX)

S2 File Abundant differential gene pathways in the negative control (NC), PC, and BL treatment groups. (DOCX)

S1 Fig Community diversity and richness in chickens in groups NC, FC and PC. Boxplots depicting the alpha diversity indexes Shannon (A), Observed Species (B), and chao1 (C). *, *P* < 0.05; **, *P* < 0.01 by Mann–Whitney U test. NC, negative control; PC, treated with *C*. *perfringens*, coccidia, and fishmeal; FC, treated with fishmeal and coccidia.
(TIF)

S2 Fig Principal coordinate analysis of the bacterial community structures in the cecal microbiota of groups NC, FC and PC. NC, negative control; PC, treated with *C*. *perfringens*, coccidia, and fishmeal; FC, treated with fishmeal and coccidia.
(TIF)

S3 Fig Venn diagram summarizing the numbers of common and unique bacterial taxa at the genus level among the three groups (FC, PC and NC). NC, negative control; PC, treated with *C*. *perfringens*, coccidia, and fishmeal; FC, treated with fishmeal and coccidia.
(TIF)
