# Supplementary material for: Disruption in the cecal microbiota of chickens challenged with Clostridium perfringens and other factors was alleviated by Bacillus licheniformis supplementation
Source: PLoS One. 2017 Aug 3;12(8):e0182426. doi: 10.1371/journal.pone.0182426 (PMC5542615; doi:10.1371/journal.pone.0182426)
Supplement: S1 Table — (DOCX) [file pone.0182426.s001.docx]

**Table S1. Composition of the diet and nutrient levels**

| Ingredient (g/kg) | Corn-soybean meal diet | High fishmeal diet |
| --- | --- | --- |
| Corn | 51.64 | 53.8 |
| Soybean (44.2% crude protein) | 39.6 | 7.44 |
| Fish meal(62.8% crude protein) | 0.0 | 30.0 |
| Colza oil | 4.3 | 4.3 |
| Dicalcium phosphate | 1.85 | 1.85 |
| Limestone | 1.3 | 1.3 |
| D,L-Methionine | 0.2 | 0.2 |
| Salt | 0.4 | 0.4 |
| Choline | 0.18 | 0.18 |
| Vitamin Premix^a^ | 0.03 | 0.03 |
| Mineral Premix^b^ | 0.5 | 0.5 |
| Nutrient Level^c^ | | |
| Crude protein | 21.17 | 25.98 |
| Metabolisable energy (MJ/kg) | 14.16 | 14.31 |
| Methionine | 0.49 | 0.95 |
| Lysine | 1.03 | 1.6 |
| Threonine | 0.77 | 0.95 |
| Calcium | 1.07 | 2.11 |
| Total phosphorous | 0.71 | 1.35 |

^a^ Vitamin Premix provided the following per kilogram of complete feed: vitamin A, 50 000 IU; vitamin D_3_, 10 000 IU; vitamin E, 25 IU; vitamin K_3_, 35 mg; vitamin B_3_, 25 mg; vitamin B_2_, 16 mg; vitamin B_6_, 6 mg; vitamin B_1_, 2 mg; vitamin B_12_, 0.03 mg; nicotinic, 25 mg; folic acid, 0.5 mg.

^b^ Mineral Premix provided the following per kilogram of basal diet: Mn (as manganese sulfate), 60.00 mg; Zinc (as zinc sulfate), 40.00 mg; Cu (as copper sulfate), 8.00 mg;Fe (as ferrous sulfate), 80.00 mg;Se (as sodium selenite), 0.15 mg; I (as potassium iodate), 0.35 mg.

^c^ Nutrient levels were calculated composition.
