# Supplementary figures and images for: Disruption in the cecal microbiota of chickens challenged with Clostridium perfringens and other factors was alleviated by Bacillus licheniformis supplementation
Source: PLoS One. 2017 Aug 3;12(8):e0182426. doi: 10.1371/journal.pone.0182426 (PMC5542615; doi:10.1371/journal.pone.0182426)

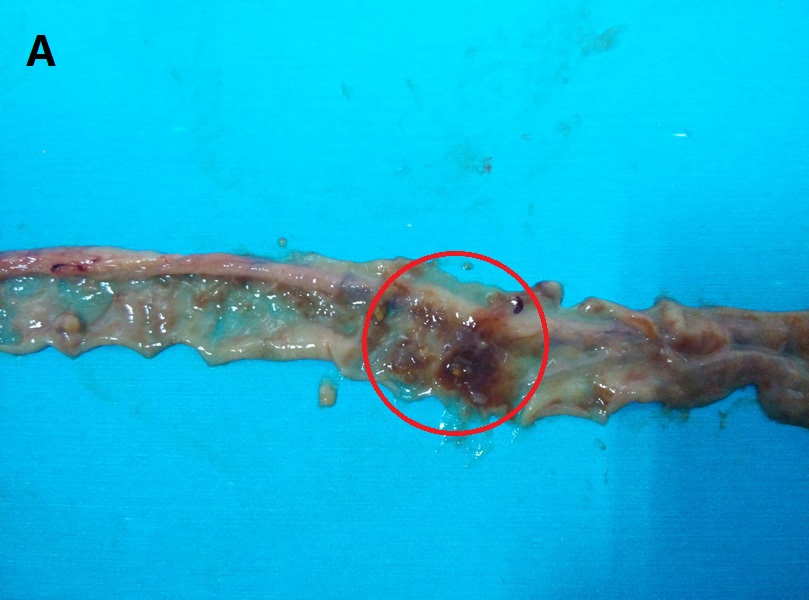


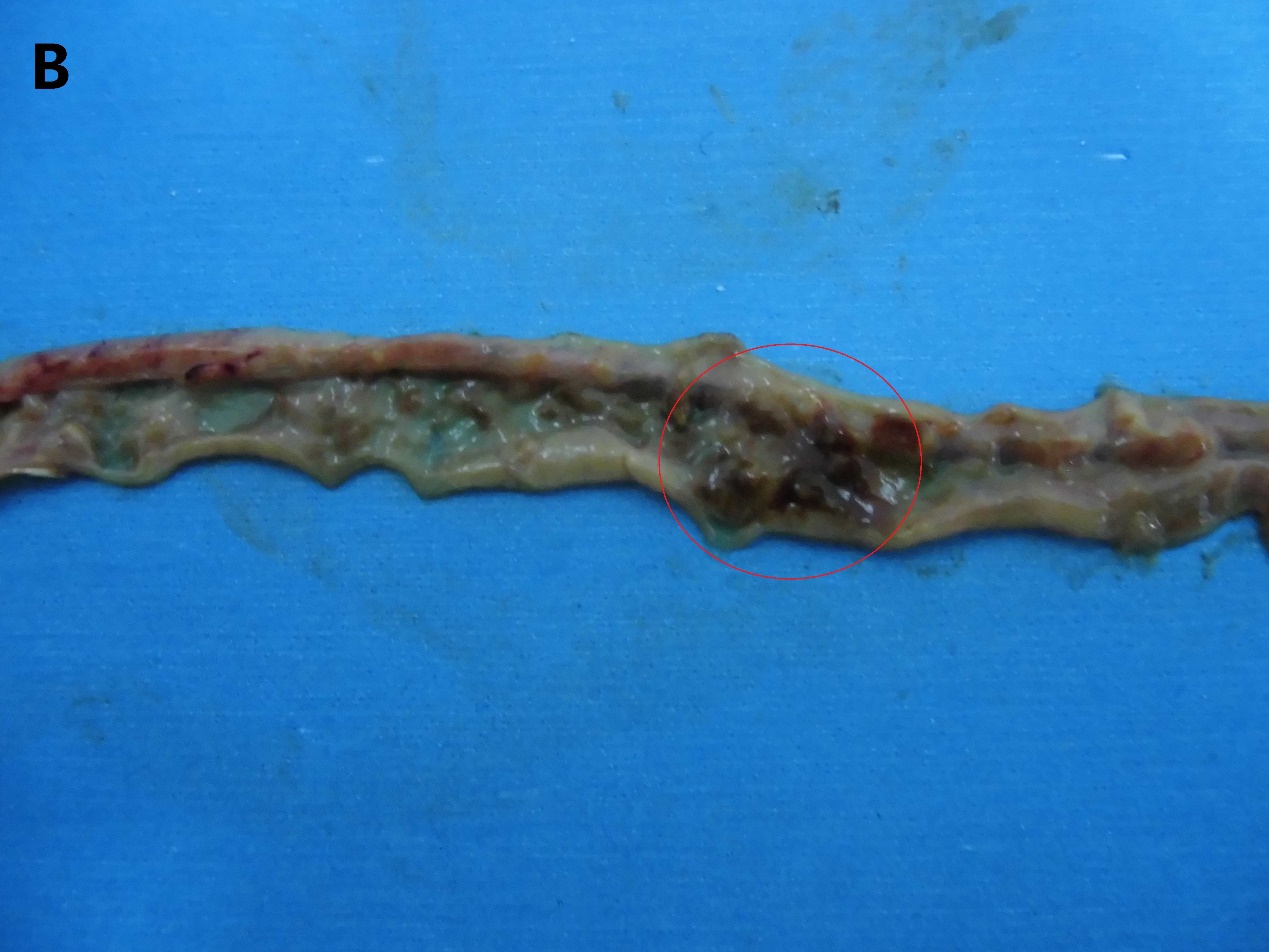


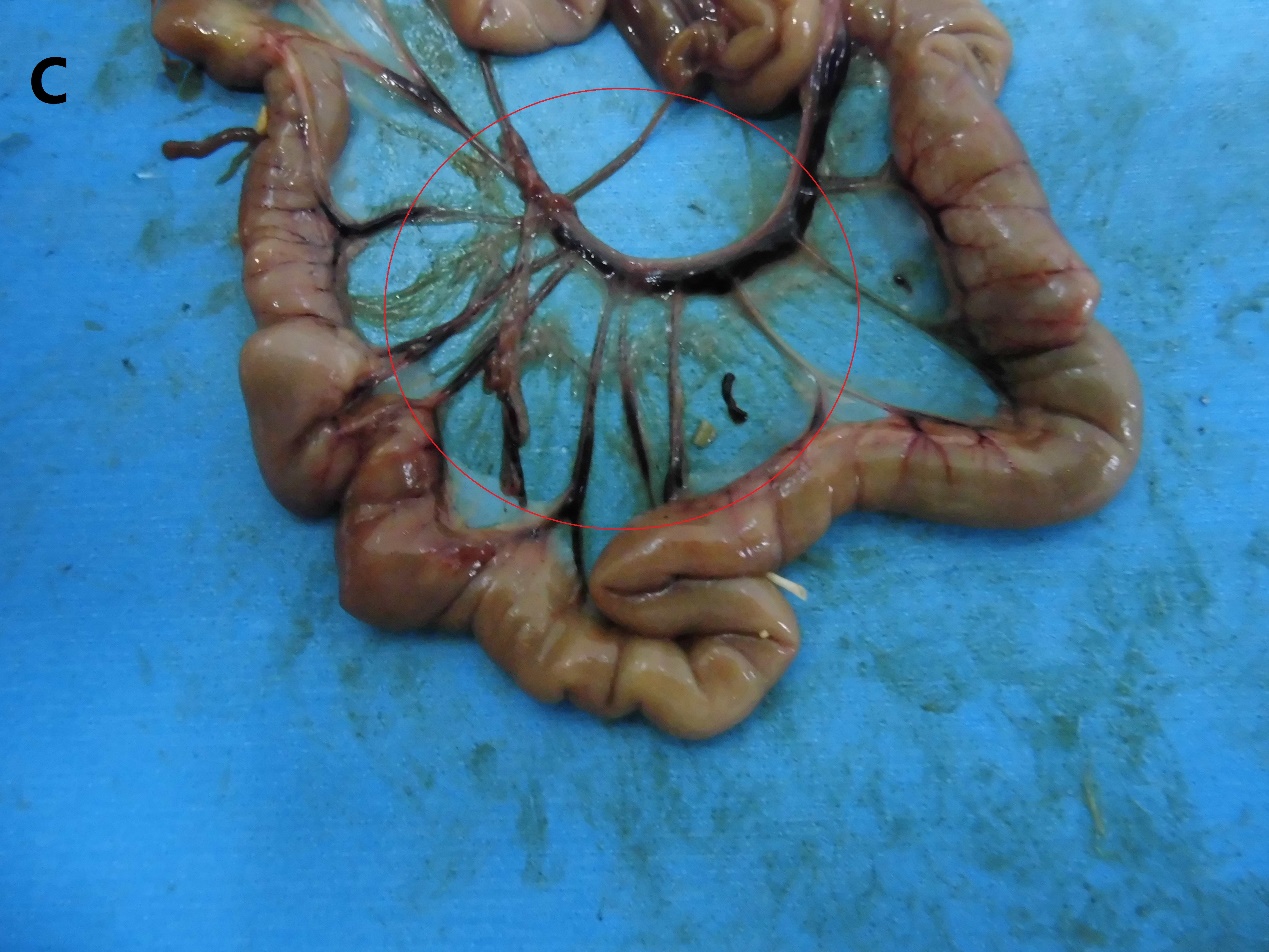

Supplement: S1 File — Large focal lesions were found on the luminal surfaces of the chickens’ small intestines (pictures A and B). We also found congestion of the mesentery (picture C). (DOCX) [file pone.0182426.s004.docx]

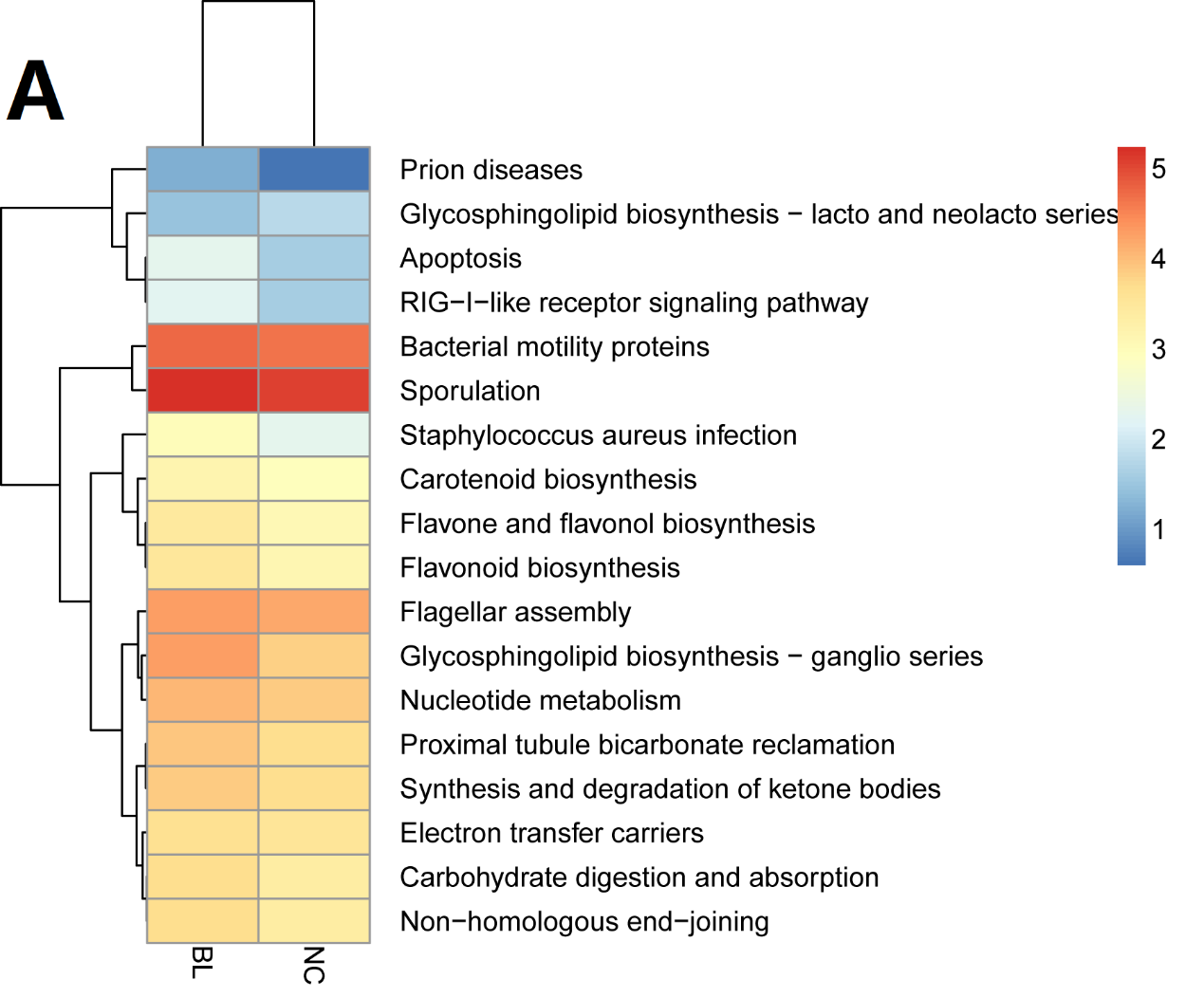


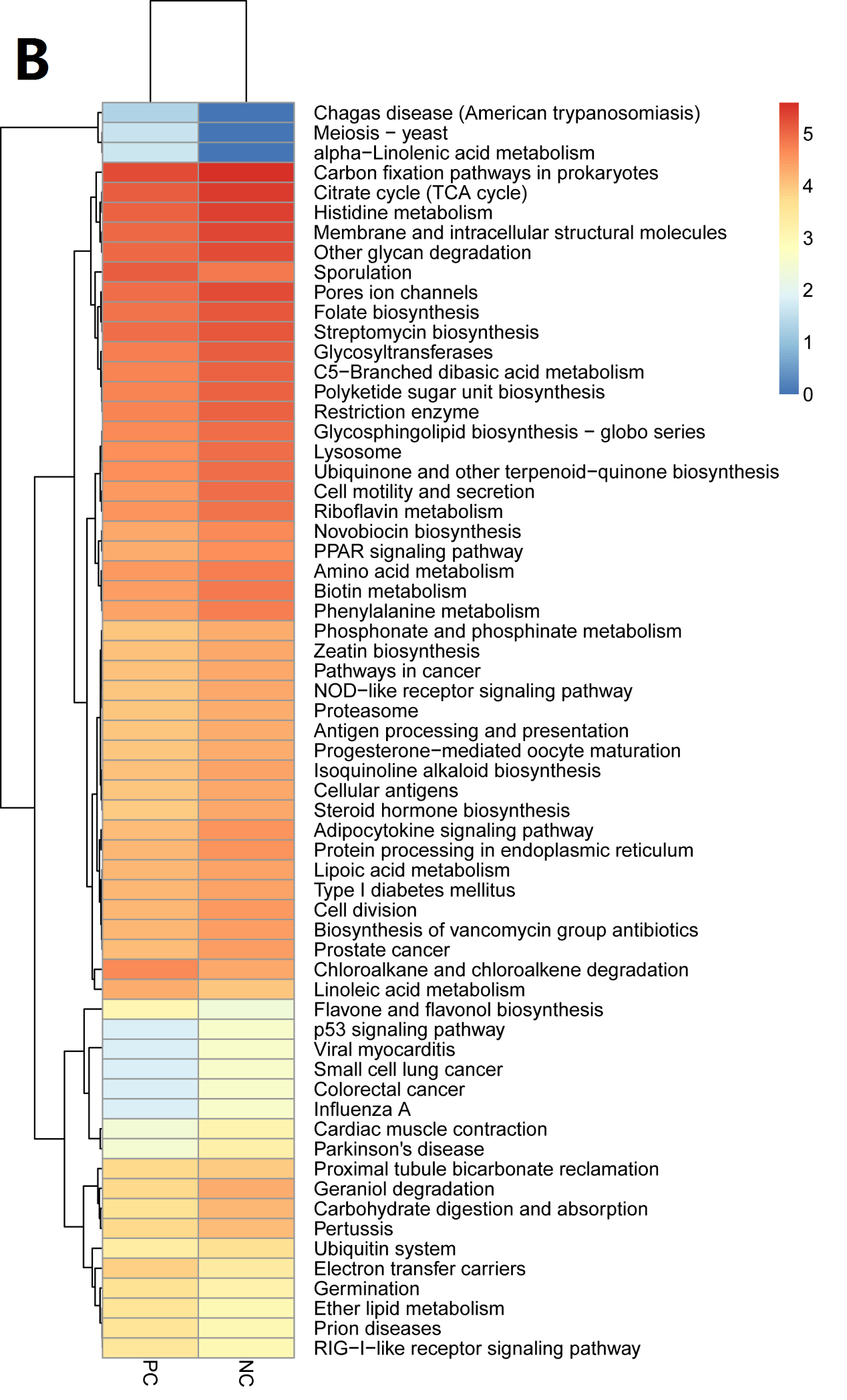


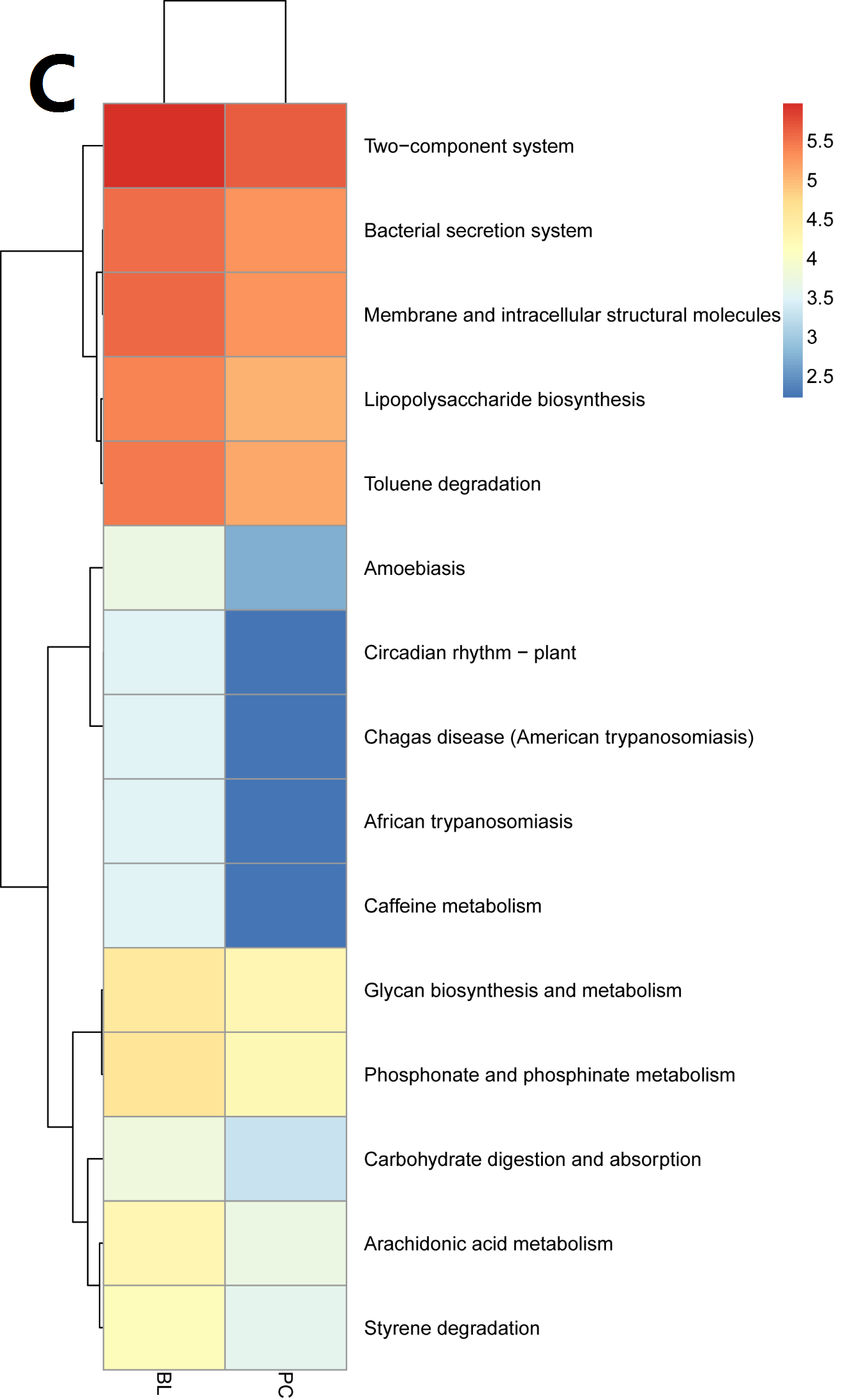

Supplement: S2 File — (DOCX) [file pone.0182426.s005.docx]

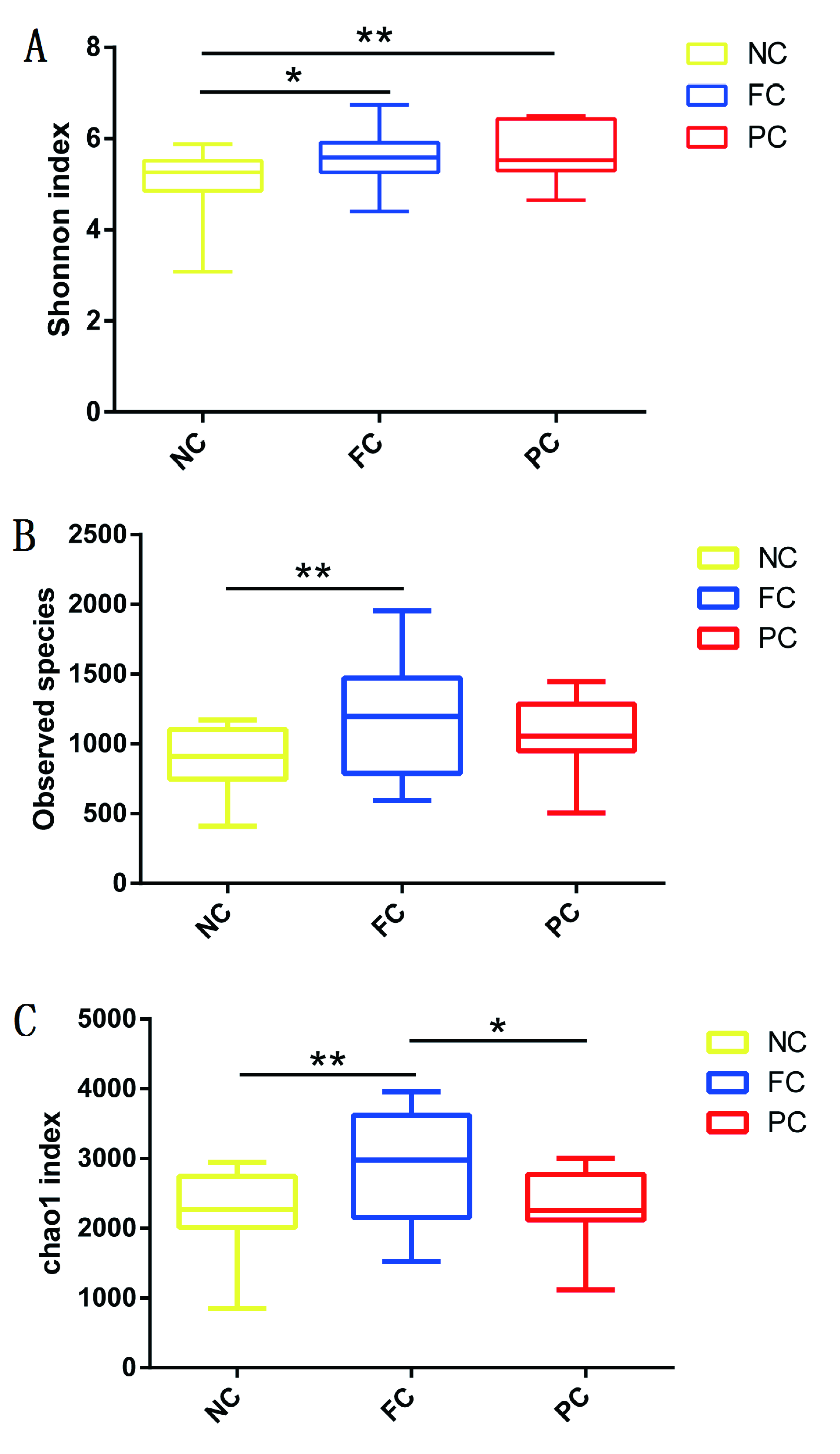

Supplement: S1 Fig — Boxplots depicting the alpha diversity indexes Shannon (A), Observed Species (B), and chao1 (C). *, P < 0.05; **, P < 0.01 by Mann–Whitney U test. NC, negative control; PC, treated with C. perfringens, coccidia, and fishmeal; FC, treated with fishmeal and coccidia. (TIF) [file pone.0182426.s006.tif]

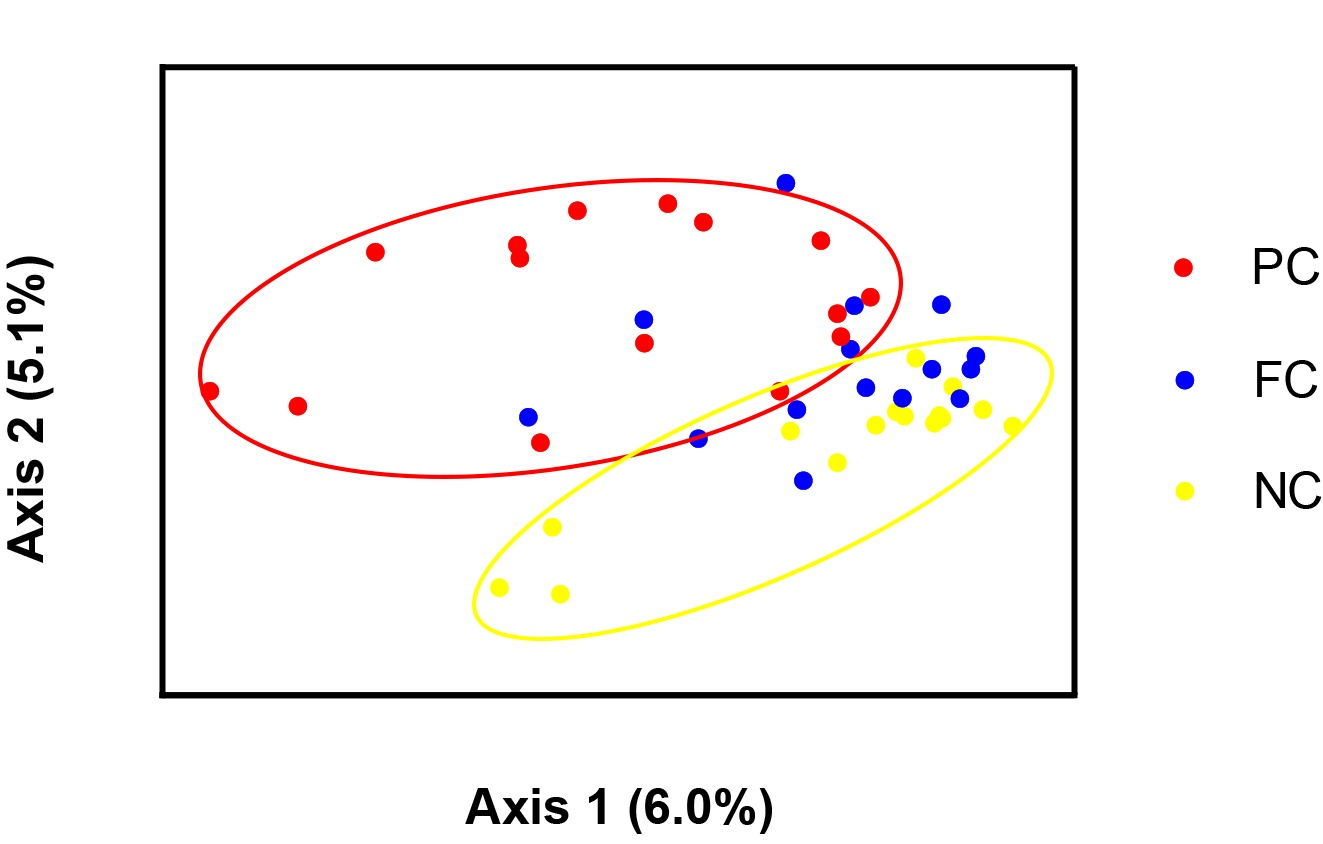

Supplement: S2 Fig — NC, negative control; PC, treated with C. perfringens, coccidia, and fishmeal; FC, treated with fishmeal and coccidia. (TIF) [file pone.0182426.s007.tif]

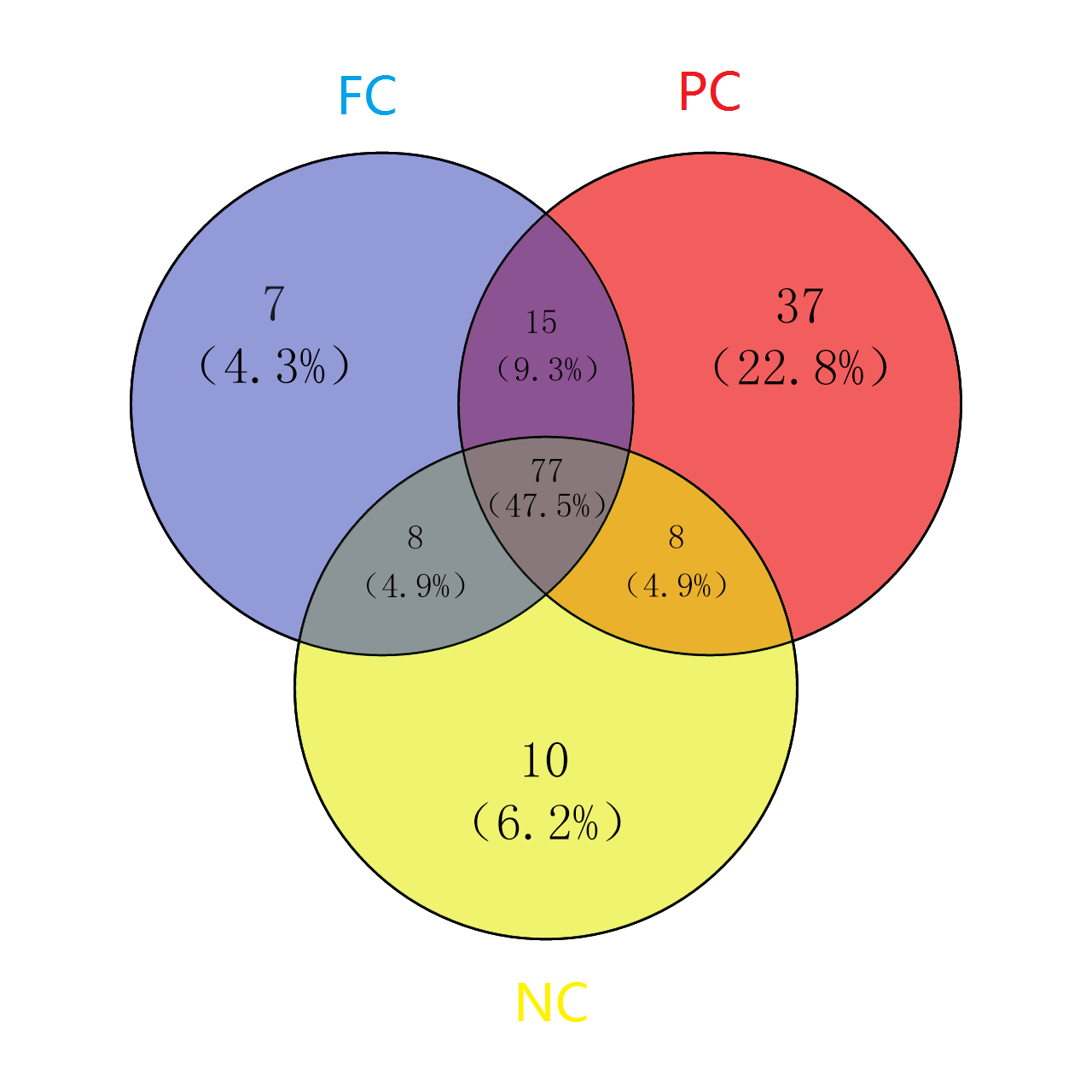

Supplement: S3 Fig — NC, negative control; PC, treated with C. perfringens, coccidia, and fishmeal; FC, treated with fishmeal and coccidia. (TIF) [file pone.0182426.s008.tif]
